# Comparison of primary and secondary stability of a one-piece compressive conometric implant system in the posterior upper and lower jaws

**DOI:** 10.3389/froh.2025.1625593

**Published:** 2025-07-25

**Authors:** Thabat R. Al-Hity, Bilal El-Dhuwaib, Abdulsalam R. Al-Zahawi, Sarhang Sarwat Gul

**Affiliations:** ^1^ Department of Maxillofacial and Oral Surgery, College of Dentistry, University of Bilad Alrafidain, Baqubah, Iraq; ^2^ Department of Maxillofacial and Oral Surgery, College of Dentistry, University of Sulaimani, Sulaymaniyah, Iraq; ^3^ Associate Dental Surgeon, Nationwide Health Care, NHS, Sheffield, United Kingdom; ^4^ Academic Unit of Restorative Dentistry, School of Clinical Dentistry, Claremont Crescent, Sheffield, United Kingdom; ^5^ Department of Conservative Dentistry, College of Dentistry, University of Sulaimani, Sulaymaniyah, Iraq; ^6^ Medical Laboratory Department, College of Health and Medical Technology, Sulaimani Polytechnic University, Sulaymaniyah, Iraq; ^7^ Department of Periodontics, College of Dentistry, University of Sulaimani, Sulaymaniyah, Iraq

**Keywords:** compressive implant, conometric, one-piece implant, primary stability, secondary stability

## Abstract

**Background:**

Implant stability plays a key role in establishing implant osseointegration and is a crucial factor in determining the appropriate timing for functional loading with a fixed prosthesis. This study aims to investigate the primary and secondary stability of a one-piece compressive conometric implant system in the posterior upper and lower jaws.

**Materials and methods:**

A total of 46 compressive one-piece dental implants (DIs) from ROOTT Trade Company were placed in the upper and lower jaws. Ready-made conometric TEC (titanium) with a prosthesis was fixed to the abutment. Implant stability was measured using an implant stability tester (AnyCheck device; NeoBiotech, Republic of Korea) by resonance frequency analysis (RFA) at facial, oral, mesial, distal, and occlusal surfaces. These measures were collected at baseline immediately after implant insertion, after 3 months, and at 6 months. The Kruskal–Wallis test was used to compare between the time points.

**Results:**

In total, 14 patients (8 men, 6 women; mean age 61.08 years; age range 35–79 years) were included. The median baseline RFA values show statistically significant differences between maxillary [55, interquartile range (IQR): 51–61] and mandibular (64, IQR: 60–67) as well as between men (59, IQR: 53–64) and women (67, IQR: 64–69). The occlusal surface shows the highest RFA compared to other surfaces. DI stability significantly increases in the maxilla and men after 6 months compared to the baseline (*p* = 0.0006 and 0.0001, respectively).

**Conclusions:**

This study suggests that the one-piece compressive conometric implant system demonstrates reliable primary and secondary stability in the posterior upper and lower jaws, with a notable improvement after 6 months.

## Introduction

Primary dental implant (DI) stability plays a major role in establishing implant osseointegration and is considered an important factor in determining the appropriate timing for functional loading with a fixed prosthesis. There are different methods to determine implant stability, such as clinically examining DI mobility using a manual instrument, insertion torque value (ITV), using an electronic instrument, and radiograph evaluation of the bone around the implant; however, researchers have found that these techniques are not sensitive enough. Thus, quantitative methods to measure DI stability have been introduced, such as the use of resonance frequency analysis (RFA) (1, 2). The RFA device (Osstell ISO; Integration Diagnostics AB, Gothenburg, Sweden) can measure DI stability with more valuable information, and its range of 1–99 with a value greater than 70 is considered to indicate higher DI stability (3–5). The damping capacity analysis (DCA) device (Periotest; Medizintectechnik Gulden, Modautal, Germany) is another non-invasive method of measuring DI stability (6). Recently, AnyCheck (Neobiotech, Seoul, Republic of Korea) was introduced as a modified DCA device to measure DI stability (again in the range of 1–99). DI stability values of 1–59 were considered low stability, values of 60–64 indicated moderate stability, and values above 65 indicated high stability (7).

Primary DI stability is achieved at the time of placement and is a mechanical phenomenon influenced by the implant design, placement technique, and the quality and quantity of the local bone. In contrast, secondary DI stability results from bone formation at the implant–tissue interface and within the surrounding bone (7, 8). In addition, the timing of DI loading—whether immediate or delayed—can impact secondary DI stability. Studies have shown that applying different approaches to immediate loading can lead to survival rates comparable with those of conventionally delayed loading (9).

Various types of prostheses are commonly retained to DIs, including removable options with precision attachments and fixed restorations retained either by cement or screws. However, both cement- and screw-retained restorations have disadvantages, including screw loosening and residual cement causing mucosal trauma (10). Conometric retention offers an alternative approach, relying on frictional contact and the elastic deformation of the coping to secure the prosthesis to the implant abutment rather than using cement or screws (11, 12).

One-piece implants with compressive threads, introduced by ROOTT Implant Company, are designed for multiple-unit restorations and can support immediate loading when adequate bone is available. Studies comparing the stability of one-piece and two-piece DIs have shown that both systems are clinically effective and offer consistent, long-term results (13). Recently, the compressive DI has been developed with conometric titanium caps (TECs) available in various sizes and lengths. However, the primary and secondary DI stability of this system has yet to be examined. Notably, the DI with TEC addresses common issues such as screw loosening and mucosal trauma caused by residual cement. Therefore, this study aimed to compare the primary and secondary stability of the compressive conometric DI (ROOTT compressive with conometric cap) in the posterior upper and lower jaws and to determine the optimal timing for functional loading.

## Materials and method

This clinical trial was registered at the Clinical Trial Registry of the U.S. National Library of Medicine (registration no. NCT06800508) under the protocol title Primary and Secondary Stability of a One-Piece Compressive Implant System (University of Sulaimani Unique Protocol ID: 227/24). All patients enrolled in the study were already seeking DIs to replace missing teeth. Participation was voluntary, and all patients provided written informed consent after reading the patient information sheet. Each participant was followed up at 3 and 6 months after implantation. Recruitment took place between January 2024 and March 2025 at a private practice in Sulaimani City, Iraq, conducted by expert clinicians in implant and fixed prosthodontics. The inclusion criteria were as follows: missing teeth for more than 2 months, a minimum 10 mm of alveolar bone height, and no pathological findings at the implant site. Patients were excluded if they met any of the following criteria: systemic diseases that could impair osseointegration (e.g. uncontrolled diabetes), radiation therapy to the head or neck region within the past 12 months, smoking more than 10 cigarettes per day, pregnancy or lactation, prior bisphosphonate therapy, history of bone grafting or socket preservation at the implant site, or active periodontal infection.

### Implant placement and functional loading protocol placement

**Figure 1:**
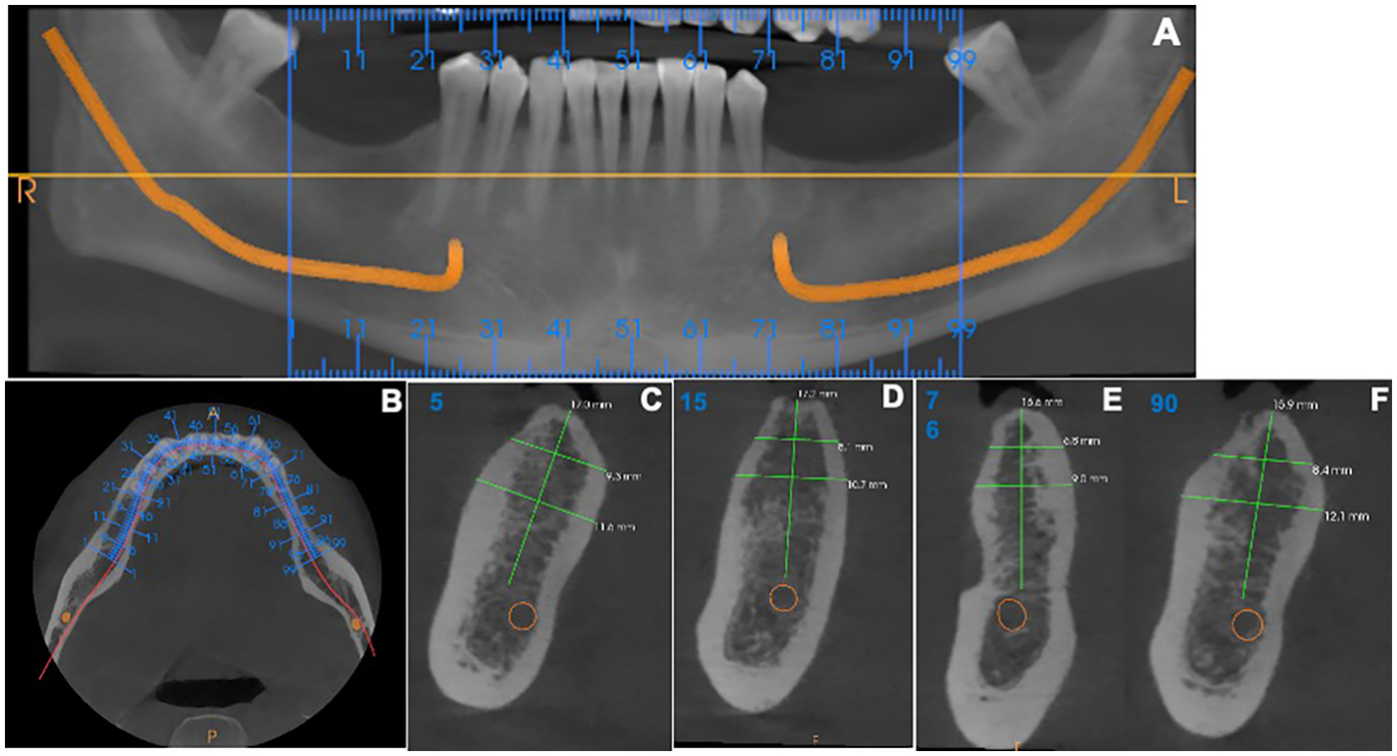
Cone-beam computed tomography images for patients included in the study. **(A)** An overview of the alveolar bone level by orthopantomography. **(B)** Axial view for the implant sites. **(C–F)** Sagittal view for measuring the alveolar bone level.

Preoperative cone-beam computed tomography was performed for all patients to assess the anatomical features of the implant sites (Figure 1). Elastomeric impressions were taken of both maxillary and mandibular arches, and study casts were fabricated using dental stone. Then, an acrylic surgical guide with a metal sleeve was fabricated to localize the implant insertion site in the jawbone without a flap.

**Figure 2:**
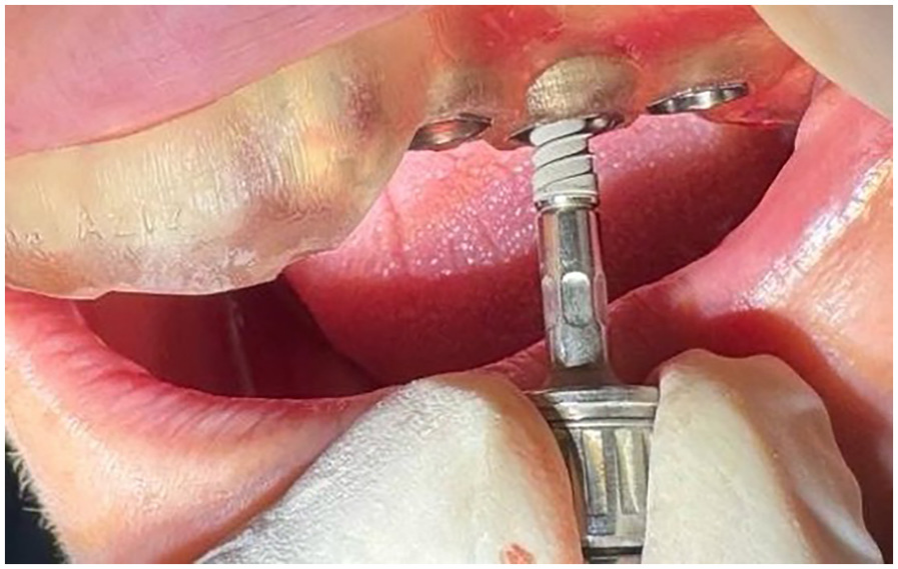
Implant insertion using a customized surgical guide.

After administration of infiltration local anesthesia, the surgical guide was positioned on the jaw and secured in place. A drilling sequence was performed according to the manufacturer's instructions to prepare a hole for implant placement (Figure 2). A compressive one-piece implant (fixture and abutment) from ROOTT Company, with a length of 10–12 mm, was inserted using a torque wrench set to 35 N/cm (14). Patients were excluded if the insertion torque was below 35 N/cm or if any loss of socket wall integrity, such as fenestration, was observed. A readymade conometric TEC (titanium) was then tapped onto the implant abutment using a small hammer until fully set. Each conometric TEC was selected, fitted, and marked on the abutment.

**Figure 3:**
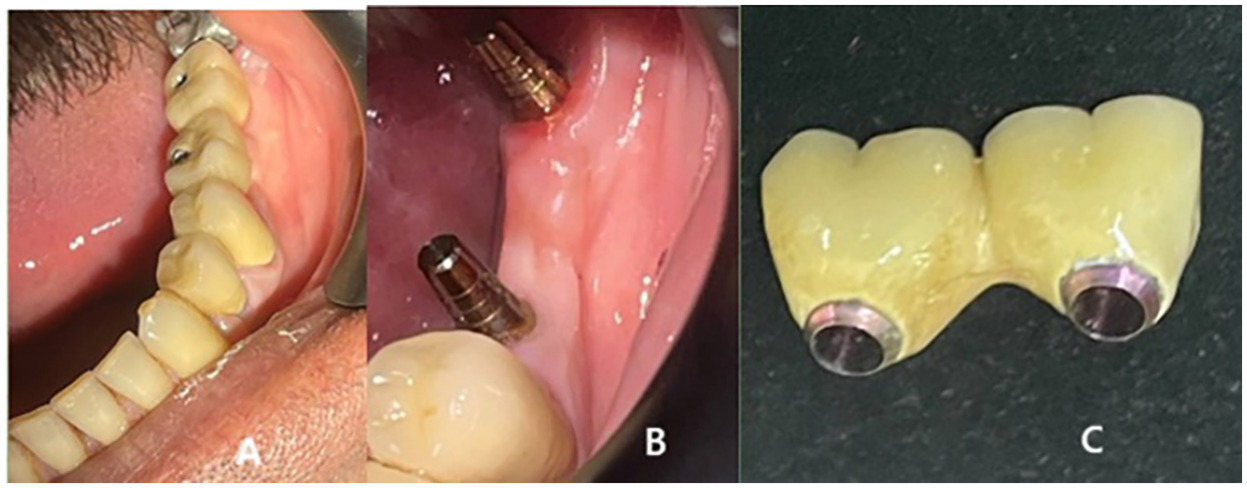
**(A)** After bridge cementation, **(B)** Removal of the bridge every time to measure the stability of the implant, and **(C)** The removed bridge.

To take an impression, a transfer cup was placed on each abutment and tied to it with soft wire to prevent it moving from its position. A one-step elastomeric heavy and light body impression was then taken, and the analog was inserted into the transfer cup. The impression and marked econometric TEC were sent to the technician for construction of the ceramic fused-to-metal bridge. After receiving the bridges and marking each conometric TEC separately within 3 days with an occlusal hole to localize the abutment, the conometric TECs were returned to the corresponding abutments marked previously. The bridges were cemented with glass-ionomer luting cement onto the retained conometric TECs fixed to the DI abutment. After the cement set, the bridge was removed from the oral cavity together with the conometric TEC as one piece using a special screwdriver inserted through the hole in the bridge, which pushed the bridge in the opposite direction to the abutment without damaging the implant. The conometric TEC, removed along with the bridge as a single unit, was cleaned by removing excess cement, polished, and reinserted with light tapping (Figure 3).

### Dental implant stability

**Figure 4:**
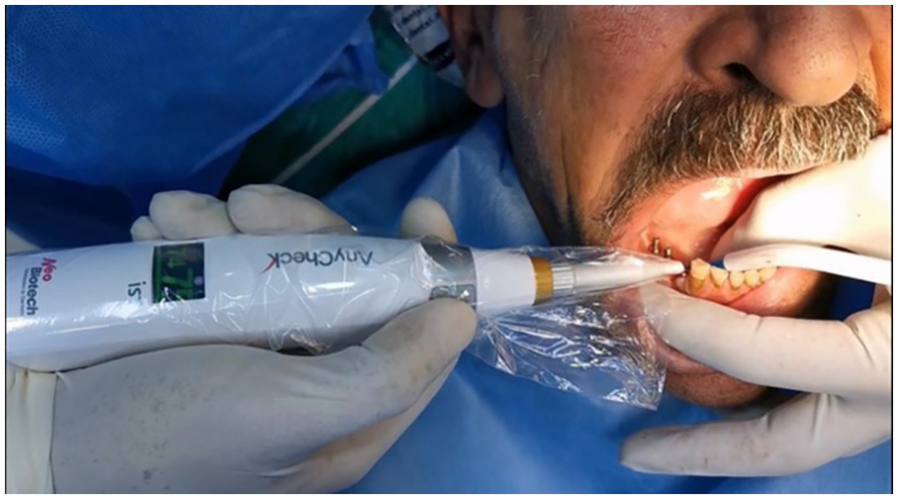
Measuring implant stability with the AnyCheck device.

DI stability was measured as RFA using an implant stability tester (AnyCheck device; Neo Biotech, Republic of Korea). Each measurement was repeated six times, following the company’s instructions that the metal rod of the AnyCheck should maintain a contact angle with the abutment within the range 0°–30° upward while measuring (Figure 4).

The first measurement (T1) was taken immediately after implant insertion and DI stability measurements were repeated after 3 months (T2) and 6 months (T3) for all five directions [buccal (B), lingual L), mesial (M), distal (D), and occlusal (O)] after removing the bridge with the driver as mentioned earlier. The bridge was then reinserted using pressure on the corresponding abutment.

### Statistical analysis

Data were assessed for normality using the Shapiro–Wilk test. The data were presented as median and interquartile range (IQR) of 25% and 75%. The Mann–Whitney *U*-test and Kruskal–Wallis test were used to compare the study's time points (T1, T2, and T3). The level of significance was set at *p* < 0.05 for the comparison of the study’s time points. All calculations were performed using SPSS software package version 26 (IBM Corp., Armonk, NY, USA).

## Results

**Figure 5:**
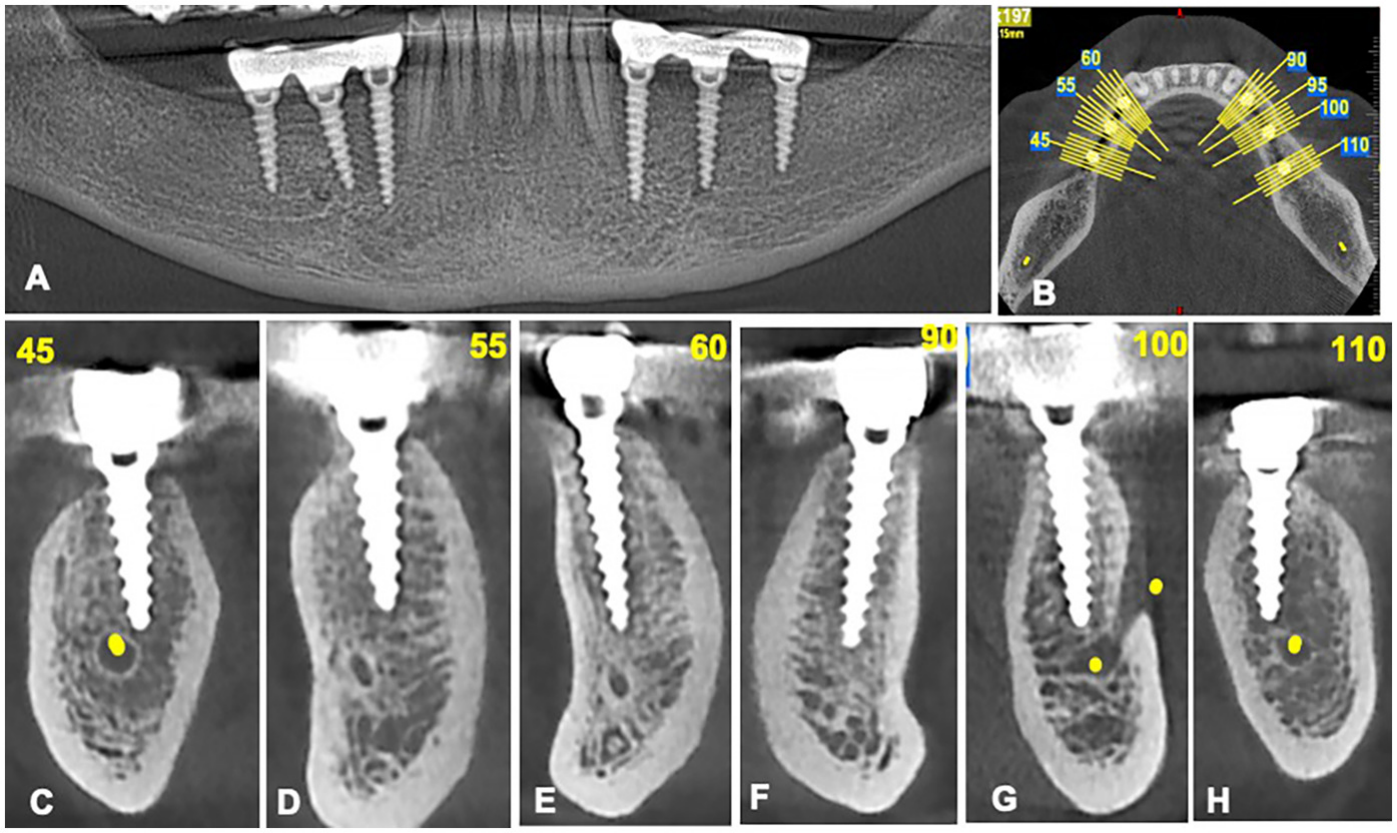
Cone-beam computed tomography images for patients after implant insertion. **(A)** An overview of the dental implants by orthopantomography. **(B)** Axial view for the implant placed. **(C–H)** Sagittal view of dental implants.

A total of 14 patients (8 men, 6 women; mean age 61.08 years; age range 35–79 years) were included in the study. Out of the 54 implants inserted, only 46 DIs, placed in the upper and lower jaws, were included in the final analysis (Figure 5). The other eight DIs were excluded from the study due to loss of integrity in the socket walls, such as fenestration and dehiscence of the supported bone and torque less than 35 N/cm. Further, participants not adhering to the study's time points were excluded.

**Figure 6:**
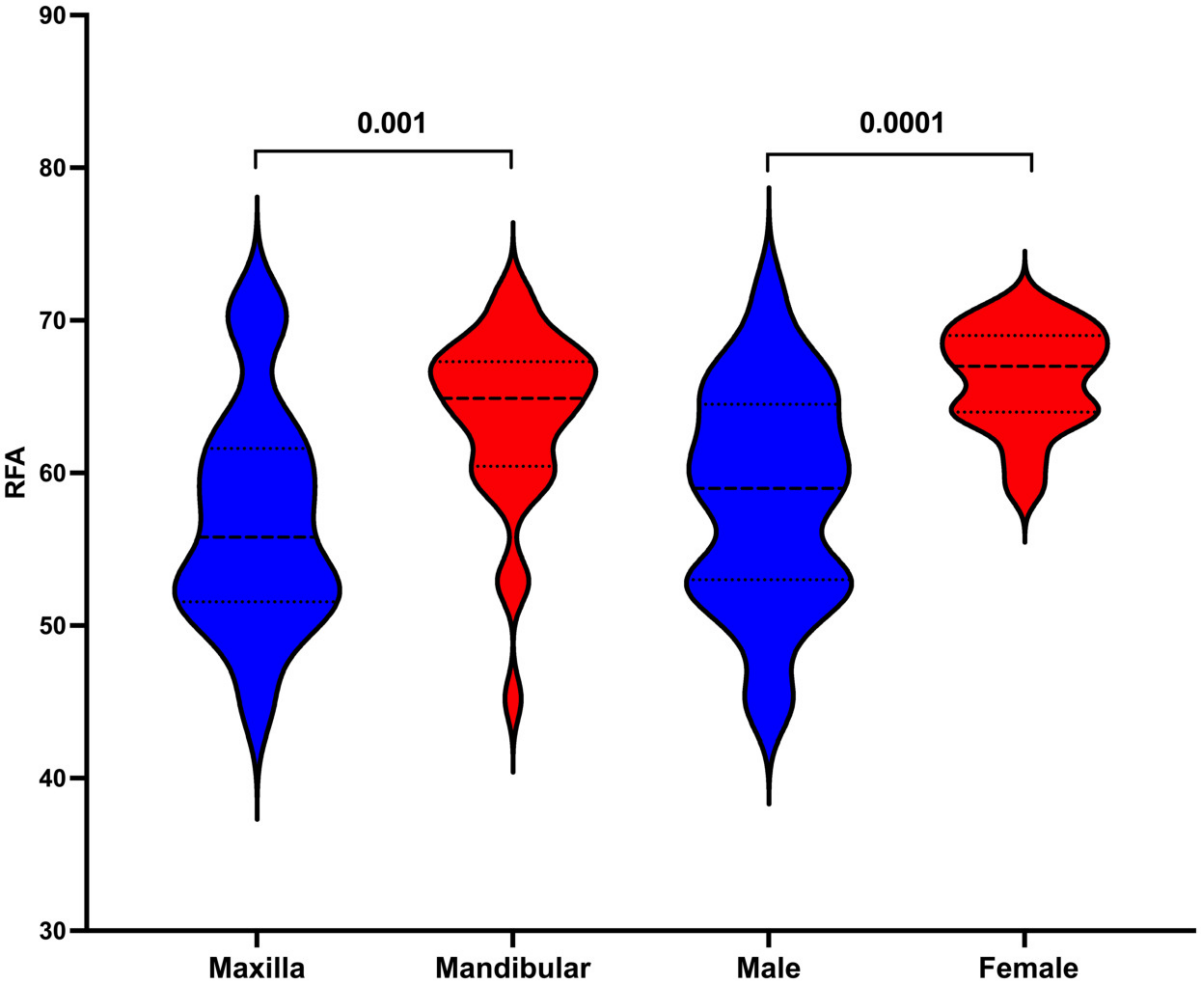
Comparison of baseline resonance frequency analysis between maxilla and mandibular as well as between men and women.

The median baseline RFA values shows statistically significant differences between maxillary (55, IQR: 51–61) and mandibular (64, IQR: 60–67) as well as between men (59, IQR: 53–64) and women (67, IQR: 64–69) (Figure 6).

Of the examined surfaces, the occlusal surface showed the highest RFA compared to other surfaces for both maxillary and mandibular DIs. Meanwhile, as mentioned earlier, the RFA in the maxilla was observed to be lower than its mandibular counterparts at the baseline time point. The RFA increased at the 3- and 6-month time points for the facial, oral, and occlusal surfaces, and these increases were statistically significant at the 6-month time point (*p* < 0.05). However, the increase in RFA for the mandibular DIs was not statistically significant (Table 1).

**Table 1:** Comparison of dental implant stability for the examined surfaces at the study's time points in maxillary and mandibular jaws.

Jaw	Surfaces	Median (IQR) RFA			Baseline vs. 3 month	3-month vs. 6 month	Baseline vs. 6 month
		Baseline	3 months				
Maxilla	Facial	45 (40–50)	49 (45–53)	59 (49–63)	0.77	0.14	0.006
	Oral	45 (42–52)	51 (48–55)	62 (53–67)	0.4	0.1	0.0009
	Mesial	54 (47–65)	55 (47–60)	65 (54–68)	0.9	0.03	0.13
	Distal	52 (43–63)	60 (52–63)	64 (59–69)	0.9	0.4	0.07
	Occlusal	79 (76–83)	82 (79–87)	86 (83–88)	0.19	0.9	0.012
Mandible	Facial	62 (55–66)	63 (52–67)	65 (59–69)	0.9	0.46	0.1
	Oral	63 (59–66)	63 (50–67)	65 (58–67)	0.9	0.9	0.9
	Mesial	59 (53–62)	63 (48–66)	62 (50–68)	0.9	0.9	0.6
	Distal	59 (50–66)	63 (45–66)	62 (48—70)	0.9	0.9	0.9
	Occlusal	83 (79–86)	82 (78–86)	85 (79–88)	0.9	0.1	0.8

Similarly, the RFA values increased at the 3- and 6-month time points compared to baseline for all examined surfaces in men. These increases at 6 months were statistically significant for all surfaces except the mesial surface (*p* < 0.05). In women, however, this increase in RFA was found not to be statistically significant at either the 3-month or 6-month time point when compared to baseline (Table 2).

**Table 2:** Comparison of dental implant stability for the examined surfaces at the study's time points in men and women.

Sex	Surfaces	Median (IQR) RFA			Baseline vs. 3 month	3 months vs. 6 months	Baseline vs. 6 months
		Baseline	3 months	6 months			
Male	Facial	50 (43–61)	54 (48–64)	63 (57–68)	0.4	0.15	0.001
	Oral	54 (44–64)	60 (50–67)	65 (57–67)	0.36	0.39	0.006
	Mesial	55 (48–59)	59 (50–66)	66 (57–69)	0.56	0.53	0.053
	Distal	53 (43–60)	60 (51–65)	63 (52–70)	0.17	0.68	0.001
	Occlusal	82 (76–86)	84 (80–87)	86 (84–88)	0.57	0.54	0.02
Female	Facial	62 (59–66)	61 (50–66)	65 (55–70)	0.9	0.64	0.9
	Oral	64 (58–65)	60 (45–65)	61 (53–68)	0.64	0.9	0.9
	Mesial	62 (59–68)	60 (45–65)	59 (50–66)	0.14	0.99	0.39
	Distal	65 (57–68)	64 (45–66)	61 (50–68)	0.9	0.9	0.9
	Occlusal	83 (76–86)	80 (69–85)	84 (79–87)	0.53	0.11	0.9

**Figure 7:**
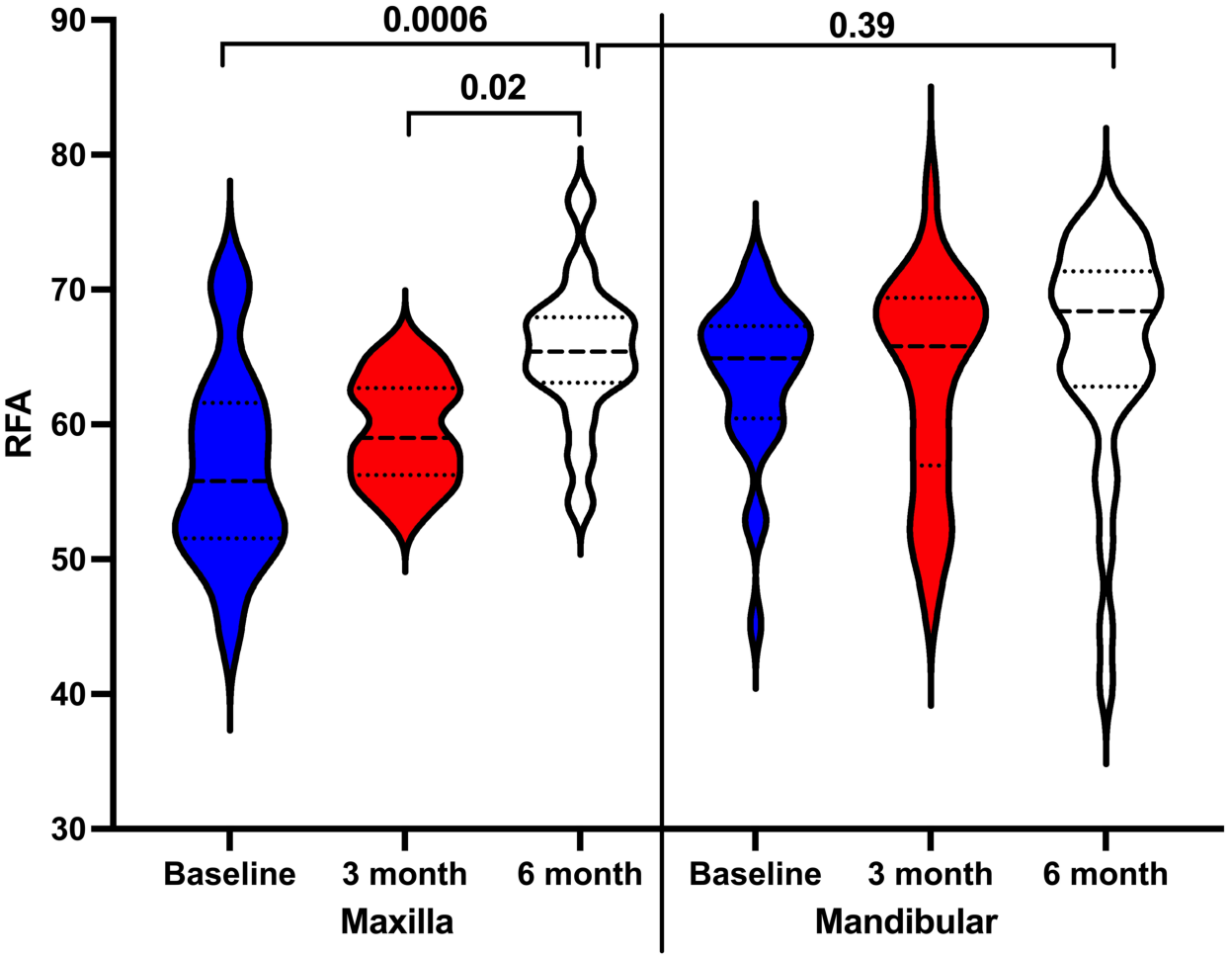
Comparison dental implant stability in the maxilla and mandibular at the study's time points.

In addition, average RFA values of all DIs were compared across the study's time points. The results showed a statistically significant increase in mean RFA values for DIs in the maxilla at 6 months (median 65, IQR: 63–67) compared to baseline (median 55, IQR: 51–61, *P* = 0.0006) and 3 months (median 59, IQR: 56–62; *p* = 0.02). Although mean RFA values in the mandible increased at both 3 months (median 65, IQR: 56–69) and 6 months (median 68, IQR: 62–71), these changes were not statistically significant compared to baseline (median 64, IQR: 60–67; *p* > 0.05). Furthermore, no statistically significant difference was found in DI stability between the maxilla and mandible at the 6-month time point (*p* = 0.39) (Figure 7).

**Figure 8:**
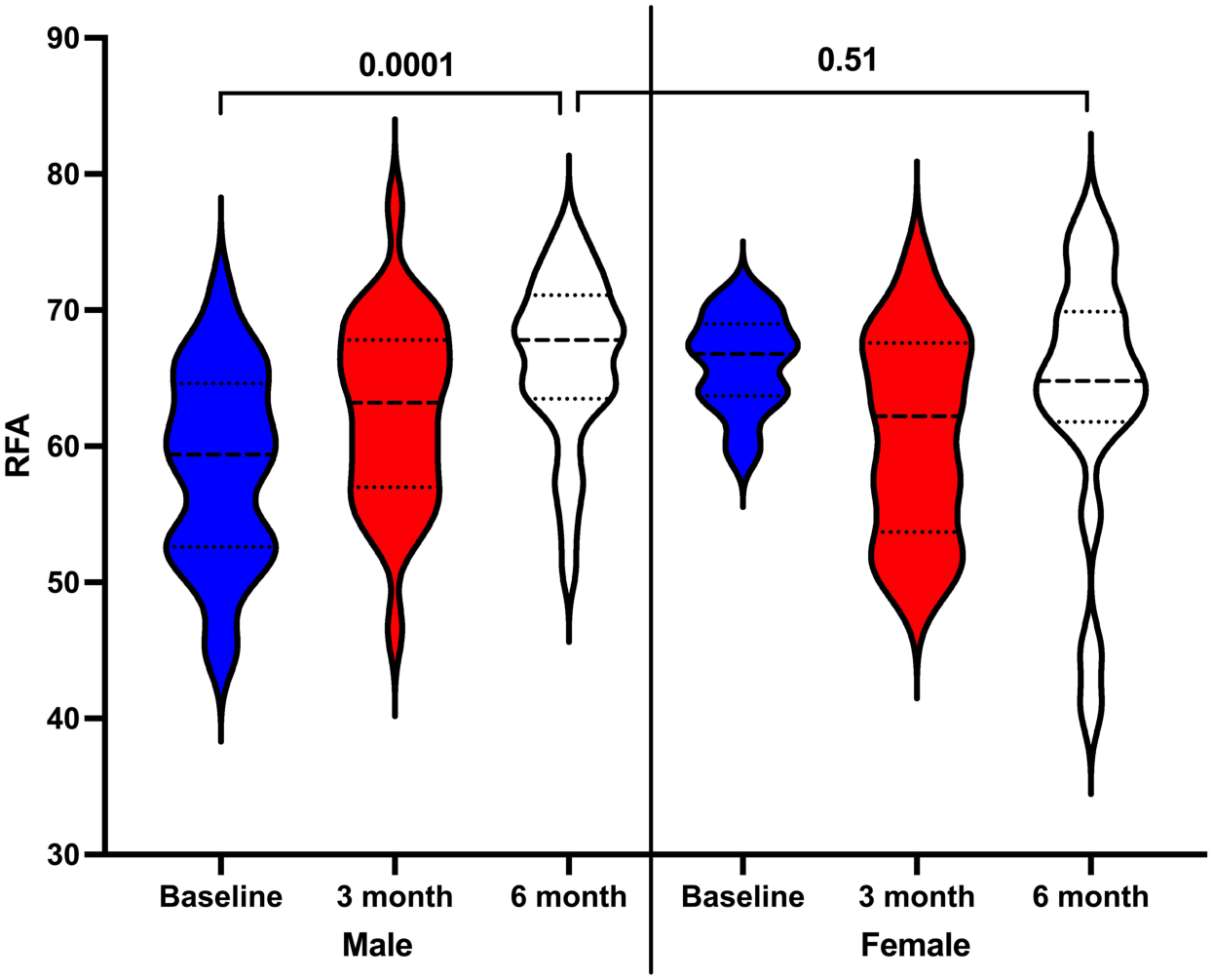
Comparison of dental implant stability in men and women at the study's time points.

On the other hand, despite increases in mean RFA values at both 3 months (median 63, IQR: 57–67) and 6 months (median 67, IQR: 63–71) in men compared to baseline (median 59, IQR: 52–64), only the 6-month RFA value showed a statistically significant increase (*p* = 0.0001). However, no statistically significant differences were found in women when comparing the 3-month (median 62, IQR: 53–67) and 6-month (median 64, IQR: 61–69) RFA values to baseline (median 66, IQR: 63–69; *p* > 0.05). Lastly, comparison of DI stability at 6 months between men and women revealed no statistically significant difference (*p* = 0.51) (Figure 8).

## Discussion

This study was conducted to investigate the effect of immediate functional loading on implant stability using a compressive one-piece dental implant retained by a conometric bridge to the fixture as an alternative to cemented and screw type. The study was based on the premise that previous results on immediate functional loading of DIs have been inconclusive (3, 13, 15–17). Furthermore, it is important to highlight that the compressive one-piece implant with immediate functional leading was introduced recently and has yet to be examined for its primary and secondary stability over time. The compressive one-piece DI system is specially designed to provide sufficient primary stability and is used in clinical situations involving horizontal alveolar ridge atrophy.

On the other hand, the conometric method applied in this study is an alternative to the cemented type, relying mainly on friction between the abutment and crown. The activation process occurs through applying a suitable insertion force for the conometric, which provides retentive performance equal to that of a cemented system without compromising mechanical functionality (11). Conometric techniques help overcome problems associated with cemented restoration, such as residual cement under the restoration leading to gingival inflammation and damage to the restoration during removal for any reason. This easy removal of the conometric-retained bridge without damaging the fixture or abutment enabled us to measure the stability of the DI separately and accurately at the study's time points.

Measuring DI stability before making a final decision for functional loading influences the DI's success and prognosis. Although both qualitative and quantitative methods are used to assess DI stability, a quantitative method was selected in this study to achieve greater precision (6, 18). It is well acknowledged that primary stability in a compression dental implant system depends on a combination of factors, including bone quality, implant design, insertion torque, placement technique, and bone–implant contact during insertion. Each factor contributes to the mechanical anchorage of the implant, which is essential for ensuring successful osseointegration in the early stages after placement (19–23).

Generally, the results of the current study showed that DI stability increases up to 6 months, likely related to osseointegration occurring over this period (24). Significantly higher stability was detected in the mandible compared to the maxilla, which may be related to bone type (type II in the mandible and type IV in the maxilla). Indeed, bone type has been proven to affect DI stability (25). It is important to note that in the current study, DI stability in the maxilla improved significantly after 6 months, with RFA comparable to that of the mandible. It has been reported that compared with delayed loading, immediate loading is associated with a higher incidence of implant failure (13). However, the immediate implant-supported conometric retention system provides fixed retention for a complete prosthesis with four implants and a 100% survival rate over 2 years.

Although our results showed higher DI stability in women compared to men, other studies found no influence of sex on DI stability (26). This can be explained by the fact the primary stability of a compressive DI system significantly relies upon the force applied during implant insertion, which creates a compression of the surrounding bone and hence increases the DI and bone surface area (27, 28). Interestingly, DI stability in men increased at the 6-month time point, matching the increase seen in women at the same time, supporting previous reports that sex does not affect secondary DI stability (29, 30).

This study has some limitations, such as a short follow-up and a lack of comparison with delayed loading or non-compressive implant systems. Nonetheless, to the best of our knowledge, it is the first study to investigate the stability of the compressive DI system with immediate loading using a conometric retainer.

## Conclusions

The results of the study indicate the reliability of both the primary and secondary stability of the one-piece compressive conometric implant system in the posterior upper and lower jaws after 6 months. In addition, secondary DI stability improved over the 6-month period. Further studies with a longer follow-up and larger sample size are needed to confirm these findings.

## Data Availability

The raw data supporting the conclusions of this article will be made available by the authors upon reasonable request.
